# Hereditary Leiomyomatosis and Renal Cell Carcinoma: A Case Report and Review of the Literature

**DOI:** 10.7759/cureus.30822

**Published:** 2022-10-29

**Authors:** Faisal A Alrawaf, Eyad S Alamri, Ali Al-Gonaim, Muneerah A Alzouman, Ahmed Alzahrani

**Affiliations:** 1 Department of Urology, King Fahad Military Medical Complex, Dhahran, SAU; 2 Department of Urology, College of Medicine, King Abdulaziz University, Jeddah, SAU; 3 College of Medicine, Prince Sattam bin Abdulaziz University, Al-Kharj, SAU; 4 Department of Pathology, Prince Sultan Military Medical City, Riyadh, SAU; 5 Department of Urology, Prince Sultan Military Medical City, Riyadh, SAU

**Keywords:** urologic cancer, urology surgery, onco-urology, laproscopic urology, endo urology

## Abstract

Hereditary leiomyomatosis and renal cell cancer (HLRCC) is a rare genetic disorder, and individuals tend to develop multiple cutaneous leiomyomas, uterine leiomyomas, and renal cell cancer (RCC).

In our study, we report the first case in Saudi Arabia, to our knowledge - a 28-year-old male with a history of right leg leiomyosarcoma post excision two years back who was referred to us with incidental finding of right kidney mass measuring 1.8x2x2.2 cm who underwent right laparoscopic radical nephrectomy, and histopathology reported it as HLRCC and RCC.

## Introduction

Hereditary leiomyomatosis and renal cell carcinoma (HLRCC) is an autosomal-dominant syndrome, and affected individuals tend to develop uterine leiomyomas, cutaneous leiomyomas, and renal cell cancer (RCC). The syndrome is caused by a heterozygous mutation in the fumarate hydratase (FH) gene, a Krebs cycle, and it’s considered a rare genetic condition [1].

The most serious manifestation of this syndrome is RCC. It is usually classified as Type II papillary RCC and frequently occurs in 10-16% of cases. Due to its early presentation at a young age and the aggressiveness of its nature more than the sporadic variety, carriers of HLRCC should be monitored closely [2].

In our study, we report the first case of HLRCC in Saudi Arabia, a 28-year-old male patient who had an incidental finding of renal mass, and later on was found to have HLRCC after radical nephrectomy.

## Case presentation

**Figure 1:**
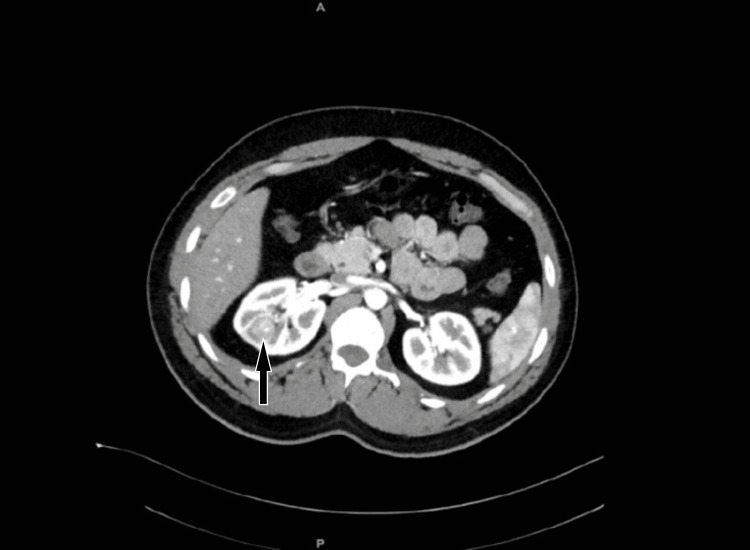
Contrast CT scan of the abdomen and pelvis, axial slice There is a right renal mid to upper pole enhancing renal mass measuring 1.8 x 2 x 2.2 cm.

**Figure 2:**
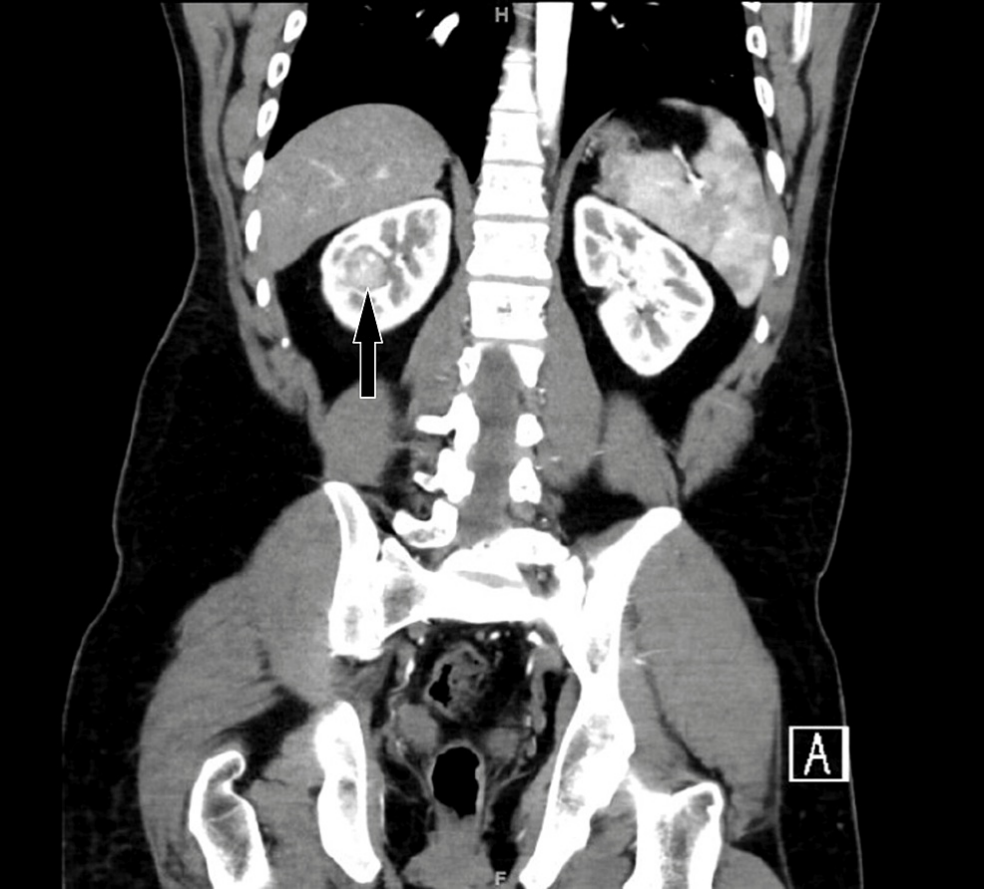
Contrast CT scan of the abdomen and pelvis, coronal slice There is a right renal mid to upper pole enhancing renal mass measuring 1.8 x 2 x 2.2 cm.

A 28-year-old male with a history of right leg leiomyosarcoma excision two years back was referred to us with incidental right kidney mass measuring 1.8x2x2.2 cm (Figures 1-2) found on PET/CT scan on his routine follow-up for leiomyosarcoma. The patient had no family history or other urological manifestation. 

**Figure 3:**
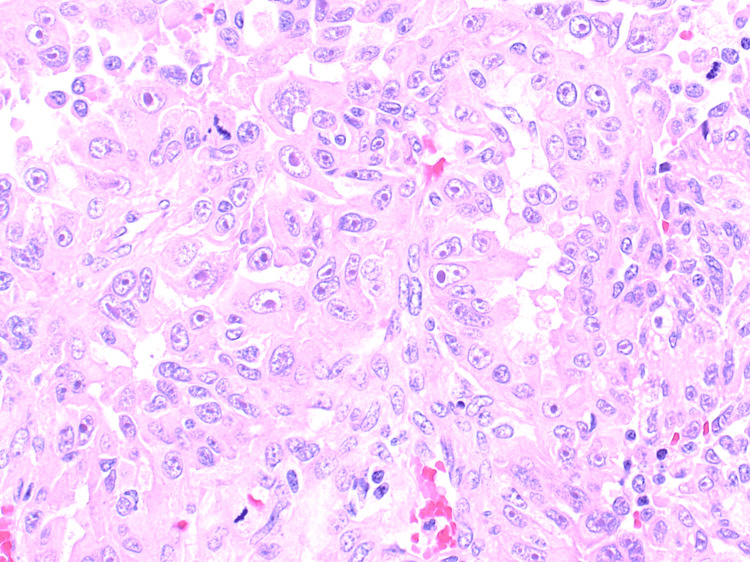
This photomicrograph (H&E, 400x) of the mass demonstrates the papillary configuration of the highly atypical tumor cells The striking inclusions like reddish macronucleoli surrounded by a clear halo are readily appreciated in this picture (arrow). In addition numerous mitotic figures are seen including an atypical form. H&E: hematoxylin and eosin

A fluorodeoxyglucose whole-body PET/CT scan was done, which showed an fluorodeoxyglucose (FDG)avid [maximum standardized uptake value​​​​​​​ (SUVmax) 13.7] 1.8x2x2.2 cm lesion in the mid pole of the right kidney. It was an enhancing heterogenous mass in the mid to upper pole of the right kidney, measuring about 1.8x2x2.2 cm, with no apparent invasion of the renal pelvis or sinus. No significant regional lymphadenopathy was noted. The left kidney appeared unremarkable. No intra-abdominal or intrathoracic metastasis was appreciated. Considering the complex nature of the mass and its intermediate complexity on RENAL nephrometry score, the patient underwent laparoscopic right radical nephrectomy. The procedure was uneventful with a unremarkable postoperative course. Histopathology reported it as a hereditary leiomyomatosis and fumarate hydratase deficient renal cell carcinoma (Figure 3).

Tumor cells were positive for AMACR while negative for CK7, CD117(c-kit), P53 and FH stain (lost). The histologic grade was G4 with lymphovascular invasion. The patient was discharged later on and placed on surveillance protocol according to his risk group (low-risk group) and was referred to the genetic disorders team for genetic testing and counseling.

## Discussion

HLRCC is considered a rare variant, with the cases known to cluster in families. Fewer than 200 cases have been reported around the world. One of the largest studies comprised 41 families [3]. Hence, genetic testing should be offered for patients with clinical presentation of HLRCC or with a family history of HLRCC because germline mutations in the fumarate hydratase (FH) gene are usually diagnostic [4]. So, the publication of such cases will increase the awareness of such rare condition.

In 2001, Launonen et al. initially described HLRCC as an autosomal dominant syndrome characterized by the development of other manifestations such as symptomatic early-onset uterine leiomyomas, cutaneous leiomyomas, and an aggressive form of type II papillary renal cell carcinoma [5].

Cutaneous leiomyoma is the most common manifestation of HLRCC, which appears in about 76-100% of patients [4]. These lesions usually appear in the second to fourth decades of life, and usually it increases in number and size with age [6]. It is usually described as a skin-colored to light brown dermal nodules, often affecting the face, neck, trunk, and extremities [2]. However such lesions were not present in our case.

A heterozygous mutation of the FH gene that encodes for an enzyme involved in the Krebs cycle, which catalyzes the conversion of fumarate to malate, is thought to be the cause of this syndrome [4].

In our case, we reported a 28-year-old male patient who had no family history of HLRCC, presented initially with right leg leiomyosarcoma, which is an unusual presentation of this syndrome, and was found to have an incidental lesion upon routine follow-up for his leg leiomyosarcoma. Natália F et al. reported a similar case, however, with a different presentation - their patient presented to the emergency room with a two-week history of right-sided flank pain and sporadic hematuria. CT showed enhancing solid tumor of 7 cm in diameter at the upper pole of the right kidney [2]. Tulandi T et al. reported a 30-year-old woman with uterine myomas, cutaneous leiomyomas, and a right renal cyst. Genetic testing showed a mutation in the patient's FH gene, however, no RCC was reported [7].

RCC is considered the most serious and severe manifestation of this syndromic spectrum, accounting for about 10-16% [6]. Compared to the sporadic form, HLRCC-associated RCCs tend to have a more aggressive behavior and present at a younger age, with a median age of detection at the age of 44 years [8].

Prognosis is mainly determined by the presence or absence of renal cancer. If the patient develops renal cancer, the risk of metastasis occurs in about 50% of the patient, and about 75% of the patients will succumb to the disease if not treated [9].

HLRCC management involves genetic counseling, symptomatic treatment, surveillance, and appropriate treatment for renal cell cancer. Genetic counseling is necessary because testing has significant ethical and personal implications for the patient, it provides certainty regarding the diagnosis and justification for kidney cancer surveillance and gives the potential for a relative's investigation [9].

Table 1 compares our case with other cases.

**Table 1:** Comparison of our case with the other cases HLRCC: hereditary leiomyomatosis and renal cell carcinoma

	Ferreira Natália et al. [2]	Togas Tulandi et al. [7]	Our case
Age at diagnosis	27	30	28
Sex	-Female	-Female	-Male
Symptoms	-Right‑sided flank pain - Sporadic hematuria	-Abdominal Pain - Abdominal swelling	-Asymptomatic
Features	-Solid tumor upper pole of the right kidney -Uterine leiomyomas - Cutaneous leiomyomas	-Right renal cyst -Uterine myomas - Cutaneous leiomyomas	-Mid pole Right kidney mass. - Leg leiomyosarcoma
Histopathology	-Papillary renal cell Carcinoma	-Not done	-HLRCC
Genetic testing	-Not conclusive	-HLRCC	-Missed follow up
Family history	-Unknown	-Positive	-Negative
Follow up	-Asymptomatic, with no evidence of cancer recurrence at 1‑year follow‑up	-Unknown	-Missed follow up.
Intervention	-Open retroperitoneal radical nephrectomy	-Myomectomy	-Laparoscopic right radical nephrectomy

## Conclusions

HLRCC is a very rare syndrome. It can be found incidentally without specific symptoms or signs, as with our case. However, some have reported different presentations, including flank pain and hematuria. Appropriate treatment is necessary to prevent the progression of this disease.
